# Supplementary material for: The Nucleotide-Binding Sites of SUR1: A Mechanistic Model
Source: Biophys J. 2015 Dec 15;109(12):2452–60. doi: 10.1016/j.bpj.2015.10.026 (PMC4699857; doi:10.1016/j.bpj.2015.10.026)
Supplement: Document S1. Two figures and one table [file mmc1.pdf]

# The nucleotide-binding sites of SUR1: a mechanistic model (Supporting Material)

Natascia Vedovato, Frances M Ashcroft and Michael C Puljung

Department of Physiology, Anatomy and Genetics

University of Oxford

Parks Road

Oxford OX1 3PT

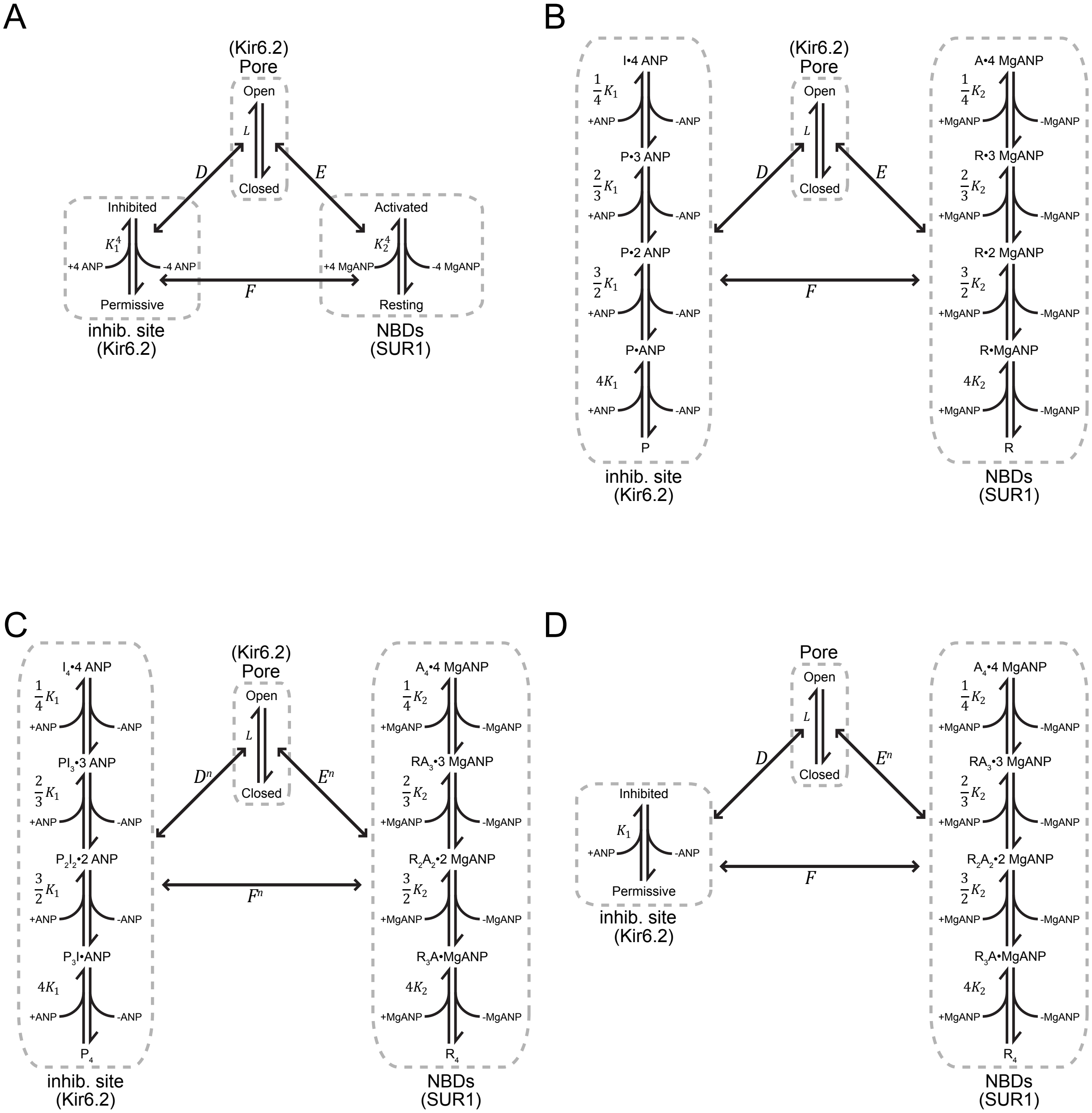

A
(Kir6.2)
Pore
Open
L
Closed
D
E
Inhibited
Permissive
+4 ANP
-4 ANP
inhib. site
(Kir6.2)
Activated
Resting
+4 MgANP
-4 MgANP
NBDs
(SUR1)
F
B
I•4 ANP
P•3 ANP
P•2 ANP
P•ANP
P
+ANP
-ANP
A•4 MgANP
R•3 MgANP
R•2 MgANP
R•MgANP
R
+MgANP
-MgANP
C
Pore
D
E
F
D

**Figure S1 Alternative gating models.** (A) Cooperative model. Schematic representing the gating of $K_{ATP}$ channels as three interacting domains, the pore, the inhibitory nucleotide-binding sites on four Kir6.2 subunits, and the NBSs of four SUR1 subunits. Nucleotide binding to SUR or Kir6.2 is cooperative and results in a concerted conformational change that affects channel gating.

$$P_o = \frac{L(K_1^4[ANP]^4D+1)(K_2^4[ANP]^4E+1)}{(K_1^4[ANP]^4+1)(K_2^4[ANP]^4+1)+L(K_1^4[ANP]^4D+1)(K_2^4[ANP]^4E+1)}$$

(B) Sequential model. Gating schematic in which nucleotides bind independently to each SUR1 or each Kir6.2 subunit. Upon binding of the fourth nucleotide to either domain, there is a concerted conformational change in all four subunits that affects channel gating. The states of the Kir6.2 binding sites are designated “P” for “permissive” and “I” for “Inhibited.” The states of the NBDs are labeled “R” for “resting” and “I” for “activated.”

$$P_o = \frac{L(K_1^4[A]^4D+4K_1^3[A]^3+6K_1^2[A]^2+4K_1[A]+1)(K_2^4[A]^4E+4K_2^3[A]^3+6K_2^2[A]^2+4K_2[A]+1)}{(K_1[A]+1)^4(K_2[A]+1)^4+L(K_1^4[A]^4D+4K_1^3[A]^3+6K_1^2[A]^2+4K_1[A]+1)(K_2^4[A]^4E+4K_2^3[A]^3+6K_2^2[A]^2+4K_2[A]+1)}$$

(C) MWC-type model. Model in which nucleotides bind to each of the four subunits of SUR1 and each of the four subunits of Kir6.2 independently and each binding event contributes energetically equally to the opening of the pore (by a factor of E for each bound SUR subunit and a factor of D for each ANP-bound Kir6.2). Subscripts indicate the number of subunits in either state.

$$P_o = \frac{L(K_1[ANP]D+1)^4(K_2[ANP]E+1)^4}{(K_1[ANP]+1)^4(K_2[ANP]+1)^4+L(K_1[ANP]D+1)^4(K_2[ANP]E+1)^4}$$

(D) Mixed model. Mixed gating model in which the NBDs each bind nucleotide independently and make equal contributions to the pore and the inhibitory Kir6.2 binding site behaves as a single site.

$$P_o = \frac{L(K_1[ANP]D+1)(K_2[ANP]E+1)^4}{(K_1[ANP]+1)(K_2[ANP]+1)^4+L(K_1[ANP]D+1)(K_2[ANP]E+1)^4}$$

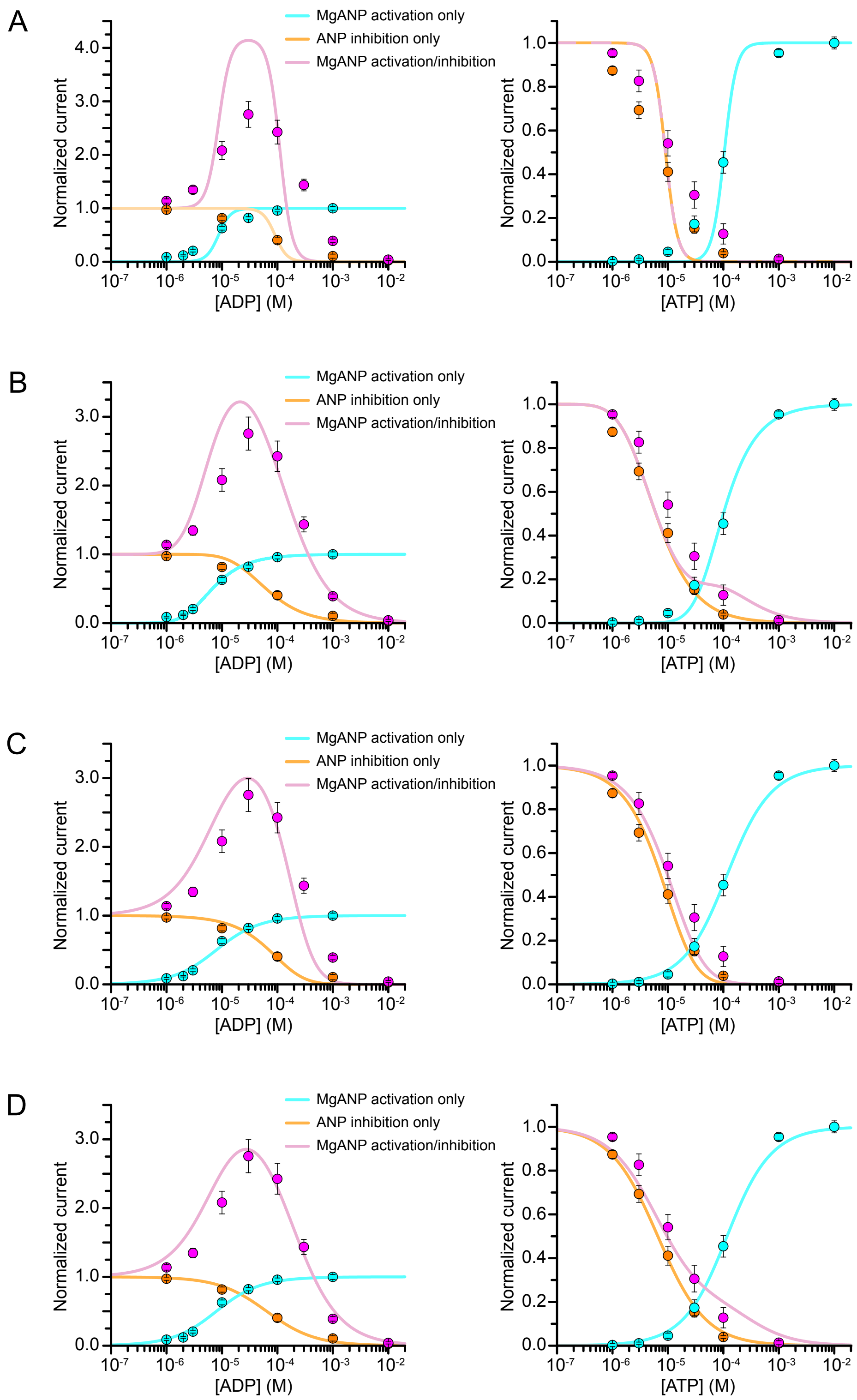

A
B
C
D
MgANP activation only
ANP inhibition only
MgANP activation/inhibition
Normalized current
[ADP] (M)
[ATP] (M)

**Figure S2 Fits to alternative gating models** (A) Fits of the cooperative scheme from Fig. S1A to data from Proks *et al.* (33). (B) Fits of the sequential scheme from Fig. S1B to data from Proks *et al.* (33). (C) Fits of the MWC-type scheme from Fig. S1C to data from Proks *et al.* (33). (D) Fits of the mixed gating scheme from Fig. S1D to data from Proks *et al.* (33). All values and RMSDs are reported in Table S1.

*Table S1 Values and RMSD for $K_{ATP}$ gating models*

| | **Single site** | | **Cooperative** | | **Sequential** | | **MWC** | | **Mixed** | |
|---|---|---|---|---|---|---|---|---|---|---|
| | **ADP** | **ATP** | **ADP** | **ATP** | **ADP** | **ATP** | **ADP** | **ATP** | **ADP** | **ATP** |
| $K_1$ ($M^{-1}$) | $1.8 \times 10^4$ | $1.8 \times 10^5$ | $1.1 \times 10^4$ | $1.2 \times 10^5$ | $9.6 \times 10^4$ | $9.9 \times 10^5$ | $3.2 \times 10^3$ | $3.0 \times 10^4$ | $1.8 \times 10^4$ | $1.8 \times 10^5$ |
| $K_2$ ($M^{-1}$) | $5.4 \times 10^4$ | $3.6 \times 10^3$ | $9.4 \times 10^4$ | $7.8 \times 10^3$ | $4.3 \times 10^5$ | $2.9 \times 10^4$ | $1.2 \times 10^5$ | $8.5 \times 10^3$ | $1.2 \times 10^5$ | $8.5 \times 10^3$ |
| $L$ | 0.18 | 0.18 | 0.18 | 0.18 | 0.18 | 0.18 | 0.18 | 0.18 | 0.18 | 0.18 |
| $D$ | $1 \times 10^{-6}$ | $1 \times 10^{-6}$ | $1 \times 10^{-6}$ | $1 \times 10^{-6}$ | $1 \times 10^{-6}$ | $1 \times 10^{-6}$ | 0.032* | 0.032* | $1 \times 10^{-6}$ | $1 \times 10^{-6}$ |
| $E$ | 9.5 | 9.5 | 9.5 | 9.5 | 9.5 | 9.5 | 1.76† | 1.76† | 1.76† | 1.76† |
| **RMSD** | 0.15 | 0.04 | 0.49 | 0.13 | 0.23 | 0.06 | 0.24 | 0.05 | 0.15 | 0.04 |

*$D^4 = 1 \times 10^{-6}$

†$E^4 = 9.5$
