# Supplementary material for: The Nucleotide-Binding Sites of SUR1: A Mechanistic Model
Source: Biophys J. 2015 Dec 15;109(12):2452–60. doi: 10.1016/j.bpj.2015.10.026 (PMC4699857; doi:10.1016/j.bpj.2015.10.026)
Supplement: Document S2. Article plus Supporting Material [file mmc2.pdf]

Biophysical Journal Volume 109 December 2015 2452–2460

Biophysical Perspective

The Nucleotide-Binding Sites of SUR1: A Mechanistic Model

Natascia Vedovato,[1] Frances M. Ashcroft,[1] and Michael C. Puljung[1,*]
[1]Department of Physiology, Anatomy and Genetics, University of Oxford, Oxford, United Kingdom

ABSTRACT ATP-sensitive potassium (K_{ATP}) channels comprise four pore-forming Kir6.2 subunits and four modulatory sulfonylurea receptor (SUR) subunits. The latter belong to the ATP-binding cassette family of transporters. K_{ATP} channels are inhibited by ATP (or ADP) binding to Kir6.2 and activated by Mg-nucleotide interactions with SUR. This dual regulation enables the K_{ATP} channel to couple the metabolic state of a cell to its electrical excitability and is crucial for the K_{ATP} channel's role in regulating insulin secretion, cardiac and neuronal excitability, and vascular tone. Here, we review the regulation of the K_{ATP} channel by adenine nucleotides and present an equilibrium allosteric model for nucleotide activation and inhibition. The model can account for many experimental observations in the literature and provides testable predictions for future experiments.

ATP-sensitive potassium (K_{ATP}) channels play important roles in a wide variety of physiological processes, including hormone secretion, neuronal function, cardiac excitability, and vascular tone (1). They comprise four Kir6.x (Kir6.1 or Kir6.2) subunits, which form a tetrameric pore, and four modulatory sulfonylurea receptor (SUR) subunits (Fig. 1 *A*). There are two SUR genes, SUR1 and SUR2, but the latter is differentially spliced to give SUR2A and SUR2B isoforms. These different SUR isoforms confer different properties upon Kir6.x.

K_{ATP} channels are modulated by numerous ligands, lipids, and drugs, but their most characteristic property is that they are regulated by cellular metabolism via changes in adenine nucleotide concentrations. Intracellular ATP and ADP cause channel inhibition (by binding to Kir6.x), whereas MgATP and MgADP stimulate channel activity by interacting with the nucleotide-binding sites (NBSs) of SUR (2–5). In addition to conferring sensitivity to Mg-nucleotide activation, SUR enhances the unliganded channel open probability (P_o) and increases the affinity for ATP block (5). It also endows the channel with sensitivity to therapeutic drugs such as sulfonylureas and K-channel openers, which inhibit and stimulate channel activity, respectively (5,6). Sulfonylureas are used to treat type 2 diabetes and neonatal diabetes (7,8), whereas the K-channel opener nicorandil is an antianginal agent (9).

Here, we briefly review our current understanding of the structure and function of the NBSs of SUR. We also provide a simple equilibrium model of nucleotide handling by the K_{ATP} complex that can account for most current data.

Submitted July 7, 2015, and accepted for publication October 22, 2015.

*Correspondence: michael.puljung@dpag.ox.ac.uk

This is an open access article under the CC BY license (http://creativecommons.org/licenses/by/4.0/).

Editor: Brian Salzberg.

© 2015 The Authors

0006-3495/15/12/2452/9

Structure of the nucleotide-binding domains

SUR belongs to the ATP-binding cassette (ABC) family of transporters (10–14). Like other ABC proteins, it consists of two sets of transmembrane domains (TMD1 and TMD2), each of which contains six helices, and two cytosolic nucleotide-binding domains (NBDs) (Fig. 1 *B*). SUR also has an additional N-terminal five transmembrane helices (TMD0). High-resolution structures of SURs or their isolated NBDs are not yet available. However, the NBSs of most ABC proteins feature a common structural fold (14). These highly conserved motifs adopt a bilobed architecture (Fig. 2). The larger lobe is a RecA-like domain that is found in other P-loop ATPases and contains the Walker A (W_A) and B (W_B) motifs, and functionally important aspartate (D-loop) and histidine (H-loop) residues. The smaller lobe, known as the α-helical subdomain, contains the ABC signature sequence (typically LSGGQ) and the Q-loop. The two lobes of the NBDs associate in an antiparallel sandwich dimer to form the two NBSs, each of which contains the W_A and W_B motifs of one NBD and the signature sequence of the other NBD. Thus, NBS1 comprises the W_A and W_B motifs of NBD1 and the signature sequence of NBD2, whereas NBS2 contains the W_A and W_B motifs of NBD2 plus the signature sequence of NBD1.

In ABC proteins, the W_A (or P-loop) motif is a phosphate-binding loop that contains a highly conserved lysine, which coordinates the β and γ phosphates of ATP. The W_B motif contains a conserved aspartate residue that coordinates Mg^{2+} and is crucial for ATP hydrolysis (14). The D-loop (containing an SALD motif) is responsible for conformational changes at the catalytic site that favor ATP hydrolysis (15). The Q-loop is approximately eight residues long, with a conserved glutamine that is thought to move into and out of the active site during the hydrolytic cycle (14). The Q-loop may also be important for the transmission of binding/hydrolysis events from the NBDs to the TMDs (16). The ABC signature sequence is located at the

http://dx.doi.org/10.1016/j.bpj.2015.10.026

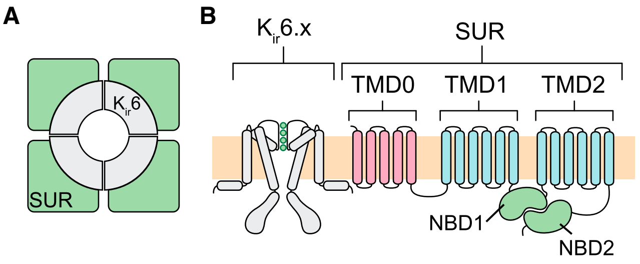

FIGURE 1 Domain organization of the K_{ATP} complex. (*A*) Hetero-octameric complex of the K_{ATP} channel, showing the Kir6.x tetrameric pore (Kir6.1 or Kir6.2) surrounded by four SUR subunits. (*B*) Schematic representation of Kir6.x and SUR protein topologies, indicating the three hydrophobic TMDs (TMD0, TMD1, and TMD2) and the two NBDs (NBD1 and NBD2) of SUR.

N-terminal end of a long helix that directs the positive end of its dipole toward the γ-phosphate of ATP (14).

SUR contains consensus (NBS2) and degenerate (NBS1) binding sites, which are so named because of the conservation of their sequences (or lack of it) compared with other ABC family members. ATP hydrolysis occurs primarily at NBS2 (17). The crystal structure of a heterodimeric ABC transporter (TM287/288) with a consensus and degenerate site has been solved (18,19). Unlike ABC proteins with two consensus NBSs (20,21), its NBDs remain in contact even in the absence of nucleotide. This dimerization is supported by a hydrogen-bonding network that mainly involves the D-loop. The D-loops also facilitate cross-communication between the two NBSs of TM287/288 throughout the transport cycle. A similar interaction appears to occur in SUR1, where the D- and H-loops are predicted to participate in formation of the NBD heterodimer. Point mutations at the predicted interface have dramatic effects on K_{ATP} channel function (22).

Clearly, a high priority in the field is to obtain an x-ray structure of the NBSs of SUR. Given that each NBS contains elements of both NBDs, crystallization of single NBDs is not sufficient. Ideally, the structure of the entire K_{ATP} channel complex would be obtained. However, this poses a considerable technical challenge. To date, only a single particle electron microscope structure has been published (23); however, its resolution is too low to permit identification of helices, let alone amino acids.

Nucleotide interactions at the NBDs

Binding studies have suggested that in addition to being structurally distinct, the two NBSs of SUR are functionally different. Covalent 8-azido-[^{32}P]ATP labeling was used to explore binding at NBS1 and NBS2 of various SUR subtypes (17,24). These studies revealed that NBS1 bound ATP in an Mg^{2+}-independent fashion, whereas nucleotide binding at NBS2 required Mg^{2+}. Furthermore, NBS1 was radiolabeled regardless of whether the ^{32}P label was on the α or γ phosphate of ATP. Only 8-azido-α-[^{32}P]ATP labeled NBS2. This suggests that NBS2, but not NBS1, hydrolyzes ATP. Detailed discussions of experiments that addressed nucleotide interactions with the NBSs of SUR can be found elsewhere (25,26).

Equilibrium model for K_{ATP} channel gating

In spite of the structural complexity of the K_{ATP} channel, with eight subunits and 12 nucleotide-binding sites (one inhibitory site on each Kir6.x subunit and two stimulatory sites on each SUR), the main features of nucleotide regulation of the K_{ATP} channel can be explained with a simplified equilibrium gating model (Fig. 3).

Our model, which is based on the gating scheme used by Horrigan and Aldrich (27) to describe activation of BK channels, considers the channel complex as three independent domains—the pore, the inhibitory site on Kir6.x, and a stimulatory site on SUR1—in coupled equilibrium (Fig. 3 *A*). The pore opens and closes in a concerted step described by the constant L (where L = [open]/[closed] = $(P_o/(1 - P_o))$ (28,29). All four inhibitory binding sites on Kir6.x are treated as a single site, which is in either an inhibited nucleotide-bound state or a permissive unbound state, as described by the affinity constant K_1 (K_1 = [Inhibited]/([Permissive][ANP]), where ANP is either ATP or

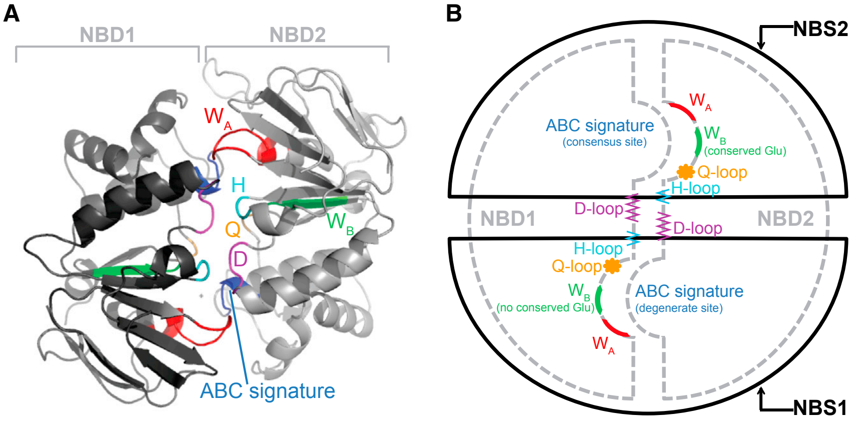

FIGURE 2 Structure of the NBSs. (*A*) Homology model of the NBSs of SUR1 based on the heteromeric structure of TM287/288 (18). (*B*) Schematic representation illustrating the functionally important regions of the NBSs.

ADP) (30,31). Likewise, all eight SUR NBSs are simplified as a single domain that exists in an unbound resting state or in an MgANP-bound active state, where K_2 = [Activated]/([Resting][MgANP]). For the purposes of this model, we have ignored the effects of PIP_2, which have been modeled elsewhere (32).

The interactions between these domains are described by three more constants: D, E, and F. D describes the allosteric interaction between the inhibitory binding site and the pore domain, and is <1. E describes the interaction between nucleotide binding at the NBDs and the pore, and is >1. F describes the direct interaction, if any, between the NBDs and the inhibitory binding site on Kir6.x. This scheme expands into the cubic model shown in Fig. 3 *B*.

To understand the contribution of the different domains to channel gating, it is necessary to isolate their individual interactions. The pore domain can be studied alone in the absence of nucleotide. The intrinsic open probability (i.e., the P_o in nucleotide-free solution) is ~40% for Kir6.2/SUR1 channels, but is subject to rundown following patch excision (33). One can isolate nucleotide inhibition at Kir6.2 by examining the inhibitory effect of nucleotide binding in the absence of Mg^{2+}, because nucleotide activation requires Mg^{2+} (2,34). When Mg-nucleotides are applied to wild-type K_{ATP} channels, they elicit a mixture of inhibition and activation. Thus, to study activation by itself, it is necessary to remove inhibition. This can be done by taking advantage of a Kir6.2 mutation (G334D) that renders Kir6.2 completely insensitive to inhibition by ATP concentrations up to 10 mM, but has a minimal effect on P_o (33,35,36). When coexpressed with SUR1, Kir6.2-G334D enables Mg-nucleotide activation to be measured directly (33).

We used the equilibrium binding scheme to fit (*solid lines*, Fig. 3 *C*) macroscopic data from Proks et al. (33). By first considering data in which nucleotides caused only activation (Kir6.2-G334D/SUR1 channels) or only inhibition (wild-type channels in Mg-free solution), we were able to greatly simplify the model. We made the assumption that the Kir6.2-G334D mutation does not influence nucleotide handling by SUR1. Additionally, we assumed the same value for L in all fits. L was calculated from a P_o of 0.15 in the absence of nucleotides. This is smaller than the P_o of 0.37 $\pm$ 0.07 reported for wild-type channels (33). However, P_o is extremely variable both between patches and between preparations, probably due to channel rundown and other variable biological processes. Furthermore, adjusting L is reasonable as this parameter primarily determines the magnitude of MgADP activation relative to the current in the absence of nucleotide, which also varies widely among different experiments (33,37–39). In terms of energetics, the difference between the measured value (0.37) and our chosen value (0.15) for P_o is <1 kcal/mol. Finally, we assumed that changes in macroscopic current were due to changes in P_o and not the number of channels (*N*). At the single-channel level, both MgADP and MgATP increased the P_o of Kir6.2-G334D/SUR1 by increasing the mean burst duration and reducing the frequency and number of interburst closed states (33).

Based on the increase in the single-channel P_o of Kir6.2-G334D/SUR1 channels elicited by saturating concentrations of MgADP and MgATP (33), which was identical for both nucleotides, we fixed the value of E, which reflects coupling of the NBDs to the pore, at 9.5. To fit the activation-only data (i.e., for Kir6.2-G334D/SUR1), we fixed K_1 at 0 to reflect a lack of binding to the inhibitory site. Therefore, the only free parameter was the affinity constant K_2. To fit the inhibition-only data (i.e., for Kir6.2/SUR1 in Mg-free solution), we fixed D at 10^{-6} because this provided the best fit to the inhibition measured experimentally at 60 mM ATP (33). K_2 was set to 0 because there is no binding to the NBDs in the absence of Mg^{2+}. Thus, the only free parameter was K_1.

We then used the values generated by fitting the activation-only or inhibition-only curves to model the response of wild-type channels to Mg-nucleotides. The only remaining free parameter was F, which describes the direct interaction between the SUR1-activation sites and the Kir6.x-inhibitory sites. We set F at 1 because varying it in either direction caused the model to deviate substantially from the data for MgADP activation.

What does the model predict?

Although the model makes several simplifying assumptions, it adequately describes the MgADP and MgATP sensitivities of Kir6.2/SUR1. We believe it may be useful as a heuristic tool to understand the gating mechanism of K_{ATP} channels. The model makes several predictions:

1) Based on both our model and the data reported in Proks et al. (33), we propose that MgADP and MgATP activate Kir6.2-G334D/SUR1 channels (and by implication Kir6.2/SUR1 channels) to the same extent. Therefore, the only difference in the activation-only curves is in the affinity constants for the two nucleotides. The predicted K_2 values are 5.7×10^4 M^{-1} (K_D = 18 μM) for MgADP and 3.93×10^3 M^{-1} (K_D = 250 μM) for MgATP. Thus, MgADP binds more tightly than MgATP to one of the NBSs of SUR1 (most likely NBS2).
2) In wild-type channels, where both activation and inhibition are observed, MgADP produces a bell-shaped concentration-response curve, with distinct activation and inhibition phases (33,37–39). In contrast, the MgATP concentration-response curve resembles that obtained for inhibition alone, with no apparent activation component but a slightly greater K_i. This implies a limited role, if any, for MgATP hydrolysis in direct channel gating. This behavior is reproduced in our model and is a consequence of the fact that MgATP has a lower affinity for

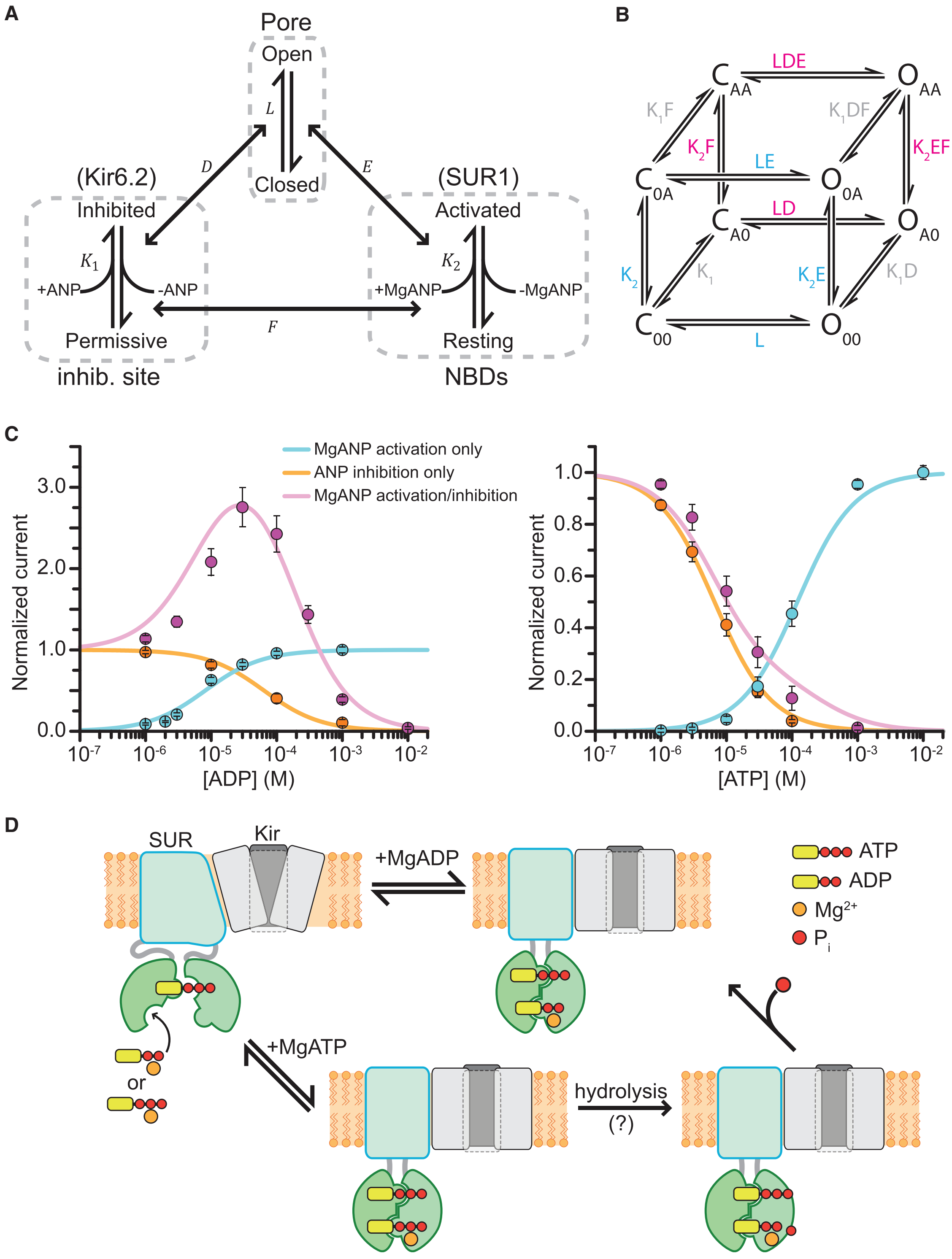

FIGURE 3 Equilibrium gating model of Kir6.2/SUR1. (*A*) Schematic representing the gating of K_{ATP} channels as three interacting domains: the pore, the inhibitory NBS on Kir6.2, and the NBSs of SUR1. (*B*) Gating scheme in (*A*) expanded into a cubic model. C and O designate the closed and open states of the pore domain, respectively. The subscripts designate the occupancy of the two NBSs as either unbound (0) or nucleotide bound (A). The first subscript refers to the inhibitory site on Kir6.2 and the second refers to the NBDs. When both inhibition and activation by nucleotides are present, the open probability (P_o) is described by

$$P_o = \frac{K_1LD + K_2LE + \frac{L}{[ANP]} + K_1K_2LDEF[ANP]}{K_1 + K_2 + K_1LD + K_2LE + \frac{1}{[ANP]} + \frac{L}{[ANP]} + K_1K_2F[ANP] + K_1K_2LDEF[ANP]},$$

(legend continued on next page)

the NBSs than does MgADP. At high nucleotide concentrations, where both the NBSs and the inhibitory binding sites are occupied, inhibition dominates because of differences in energetics. Based on the Proks et al. (33) data and the fits from our model, nucleotide binding to the inhibitory site stabilizes the closed state by ~8 kcal/mol, whereas binding to the NBSs only contributes ~1.3 kcal/mol to stabilize the open state.

3) The allosteric coupling between the NBSs and the inhibitory Kir6.2 binding site can be adequately explained by the coupling of both domains to the pore, without the need to invoke a direct, functional interaction between the two (i.e., $F = 1$). In other words, nucleotide binding to SUR does not alter nucleotide binding to Kir6.2 (and vice versa) except via changes in channel gating.
4) Hydrolysis of ATP at the NBDs is not required to explain the differences in gating between ATP and ADP. Our equilibrium model adequately represents the activation of Kir6.2/SUR1 channels without including any irreversible hydrolysis steps. This suggests that binding of MgATP (like MgADP) at NBS2 may directly promote channel opening in Kir6.2/SUR1 channels.
5) In almost all studies published to date, the experimental data were normalized in various ways to correct for rundown, and we have taken this into account in our modeling. If the rundown could be stabilized, however, and the data expressed as actual P_o rather than normalized P_o, the concentration-response curves for nucleotide effects on macroscopic currents would contain information regarding not only the concentration range over which nucleotides have their effects but also the energetic contributions that nucleotide activation or inhibition make to gating. This information would provide greater insight into the mechanism(s) underlying nucleotide modulation and the effects of disease-causing mutations in K_{ATP} channel subunits.

How valid are the model assumptions/predictions?

The main predictions of our model as they relate to Mg-nucleotide activation (as in Kir6.2-G334D/SUR1 channels) are shown in Fig. 3 *D*. As noted above, we made several simplifying assumptions. Chief among these was to treat the eight NBSs as a single unit with a single binding affinity constant. However, the reported number of SUR subunits required to activate K_{ATP} varies in the literature (40–42). We also modeled the inhibitory ATP binding sites as a single site, but this too is controversial (28,31). Therefore, we also considered four alternatives (Fig. S1 in the Supporting Material). We modeled the NBSs as four separate subunits with cooperative binding and a concerted conformational change (Fig. S1 *A*), four subunits with independent binding and a concerted conformational change when all subunits are occupied (Fig. S1 *B*), and a Monod-Wyman-Changeux-type model in which binding to each subunit is independent and each subunit makes an identical energetic contribution to the pore domain (Fig. S1 *C*). We modeled the inhibitory Kir6.2 binding sites in the same way in each of these three models. We also fit the data with a mixed model in which the inhibitory Kir6.2 binding site was a single site and the binding sites on SUR1 were treated as four individual subunits, each with independent binding and gating contributions (Fig. S1 *D*). None of the alternative models fit the data sets as well as the model presented in Fig. 3 (compare Fig. S2, *A–C*, and Table S1), with the exception of the mixed model, which did not improve upon the fit to our initial model (compare Figs. 3 *C*, S2 *D*, and Table S1). None of the fits were improved by introducing terms to describe a direct interaction between the NBDs and the inhibitory binding sites (F).

The largest deviation between our simple allosteric model (Fig. 3) and the data from Proks et al. (33) is in the fit to wild-type channels at lower MgADP concentrations (1–30 μM), where activation dominates. Importantly, none of the alternative models improved on the fit over this concentration range. Therefore, the relatively poor agreement with the data is not a consequence of the simplifying assumption that the NBDs are treated as a single binding site. This deviation may instead challenge our assumption that there is no difference in the ability of SUR1 to stabilize the open state of the channel in wild-type versus Kir6.2-G334D channels. Alternatively, it may simply result from biological variability across experiments (33,37–39).

As noted above, none of our fits had more than one free parameter. However, the values generated are only as accurate as our initial assumptions regarding L, D, and E. The value for E calculated from the single-channel open probability of Kir6.2-G334D/SUR1 channels in the absence and

where [ANP] is the nucleotide concentration and all other symbols are as described in the main text. (*C*) Cubic model (*solid lines*) fit to the nucleotide activation/inhibition data (*open circles*) from Proks et al. (33). Because the experimental data were normalized, the model was also normalized. For activation curves, this was achieved by subtracting the P_o at 0 nucleotide $(L/(L + 1))$ and dividing the difference by the maximum P_o $(EL/(EL + 1))$ minus the unliganded P_o. For inhibition curves, the equation was divided by the P_o in the absence of nucleotide $(L/(L + 1))$. When both activation and inhibition were present, the model was normalized to the P_o in the absence of nucleotide. (*D*) Simplified scheme for K_{ATP} activation via Mg-nucleotide binding at SUR1. This schematic assumes that SUR1 can hydrolyze MgATP, but that hydrolysis is not necessary for channel activation by MgATP.

presence of Mg-nucleotides was 9.5 ± 1 (± SEM). We found that varying E between 8.5 and 10.5 (equivalent to E ± SEM) only affected the value generated for K_2 by 7%. Changing the value of D by 100-fold had only minor effects (<<1%) on the K_1 values generated. Changing our estimate for P_o (and therefore L) to that reported for wild-type Kir6.2/SUR1 channels in the absence of nucleotide (0.37) had more significant effects, increasing our estimates of K_1 by 35% and decreasing the K_2 values generated from our fits by a factor of 1.8. Therefore, although we believe the major predictions of our model to be robust, some caution should be used in trusting the absolute value of the binding affinities it produces, unless P_o can be closely monitored or controlled throughout an experiment.

What is the role of NBS1?

Our model does not discriminate between Mg-nucleotide binding to NBS1 and NBS2, but it has been proposed that nucleotide interactions with NBS2 primarily drive channel opening (3,43,44). What, then, is the role of NBS1?

A solely structural role is suggested by CFTR, an ABC transporter that is also a Cl^--permeable ion channel. As is the case in SUR1, NBS1 of CFTR is degenerate. In CFTR, channel opening is initiated by binding of MgATP to NBS2 and terminated by ATP hydrolysis at NBS2 (45). In contrast, ATP stays bound to NBS1 for several gating cycles (46,47). Thus, bound ATP at NBS1 of CFTR can be considered a structural element that allows for dimerization of the NBDs when NBS2 is occupied (48).

In contrast, the functional effect of nucleotide binding to NBS1 of SUR1 is not completely understood. In part, this is because no measurements of equilibrium nucleotide binding exist. It is uncertain whether mutations in NBS1 influence ATP binding (as they do 8-azido-[^{32}P]ATP binding) (49), and SUR2A appears to handle nucleotides differently from SUR1 (or SUR2B) (50–52). Furthermore, ATP hydrolysis properties differ when measured for isolated NBDs, SUR, or the whole K_{ATP} channel complex (23,53). Thus, extrapolation from data obtained in isolated domains or subunits to K_{ATP} channel gating has to be treated cautiously.

Results from analyses of NBS1 mutations are inconclusive. Mutation of the Walker A lysine in NBS1 (K719 in SUR1) reduced high-affinity labeling of 8-azido-[α-^{32}P]ATP (49), but it is unclear whether the same is true of ATP binding. It is possible that this mutation does not alter ATP binding, as it decreased ATP hydrolysis by SUR1 without affecting the K_m for ATP (53) and reduced MgATP activation without affecting the EC_{50} (39). Although the K719A mutation strikingly shifted the EC_{50} for MgADP activation (39), this may have been mediated via an allosteric effect on NBS2, perhaps by preventing NBD dimerization. Complete deletion of NBD1 produced channels that were not activated by MgADP and were either less stable at the plasma membrane or not correctly trafficked (54). On balance, the available data suggest that NBD1 of SUR1 has an important structural role, but its influence on channel activity remains obscure. If it is indeed merely a necessary structural element, then what is being measured in experiments, and in our model, may be occupancy of NBS2.

Interestingly, mutation of the Walker A lysine (K707A) of NBS1 of SUR2A had no effect on Mg-nucleotide activation (52), which may support the idea that, in this case at least, ATP handling at NBS1 does not influence channel activity. Why the same mutation in SUR2B, which differs from SUR2A only in its last 42 amino acids, prevents MgADP activation is a puzzle.

Is MgATP hydrolysis necessary for activation?

In our model, hydrolysis of MgATP is not required for channel activation, implying that both MgADP and MgATP can promote channel activation simply by binding more tightly to the active conformation of the NBDs. The act of hydrolysis per se is not necessary to promote channel opening, as MgADP is able to activate. However, it remains a possibility that MgATP must first be hydrolyzed to MgADP to activate channels.

Numerous studies have shown that isolated NBD2 (and to a lesser extent NBD1) of SUR1 and SUR2 can hydrolyze ATP (25,43,50,53), as can SUR1 (53) and Kir6.2/SUR1 (23). In the case of Kir6.2/SUR2A, conditions that lock the channel in a posthydrolytic state favor channel opening (43). Orthovanadate, which occupies the same position as the γ-phosphate of ATP, inhibits ATPase activity and stimulates channel opening in the presence of ATP. This suggests that the MgADP·Pi state is able to stabilize channel opening. Likewise, MgADP is able to stimulate channel opening. It is worth noting, however, that the orthovanadate effect develops much more slowly than direct activation of Kir6.2-G334D/SUR1 by MgATP or MgADP. BeF, which arrests the ATPase cycle in a prehydrolytic configuration, impairs the ATPase activity of SUR1 and SUR2 (53,55) and reduces Kir6.2/SUR2A channel opening produced by MgADP (43). This suggests that the MgADP·BeF state, analogously to a prehydrolytic ATP-bound state, does not induce channel activity, but it does not necessarily imply that MgATP is unable to directly stimulate channel opening, perhaps by stabilizing a state that resembles a posthydrolytic conformation of the NBDs.

Mutation of the catalytic residues of NBS2 of SUR1 impairs ATPase activity (53). If these mutations only prevent hydrolysis, they would be expected to affect activation by MgATP, but not MgADP. However, mutation of the Walker A lysine or Walker B aspartate in NBS2 of SUR1 impairs activation by both MgADP and MgATP (2,4,34); indeed, it appears to have a larger effect on MgADP activation (39). This raises the possibility that these mutations affect either nucleotide binding directly or the conformational

change that couples binding to activation. Thus, the catalytic residues in the NBDs of SUR may have functional effects beyond MgATP hydrolysis. Experiments in which SUR1 was expressed without Kir6.2 showed that binding of ATP (or ATP analogs) under nonhydrolytic conditions (e.g., no Mg^{2+}, catalytically dead mutations) was still competent to cause a conformational change in SUR1 as assayed by a change in glibenclamide affinity (56,57). Therefore, although it seems likely that K_{ATP} channels can hydrolyze MgATP, this may not be an absolute requirement for channel activation via SUR. In future studies, single-channel and kinetic analyses could be used to identify K_{ATP} gating transitions that are not at equilibrium (e.g., irreversible hydrolysis steps), as has been demonstrated for CFTR (45).

Interactions between nucleotide binding at SUR and Kir6.x

The final question is whether or not there is a direct, functional interaction between the NBDs of SUR1 and the inhibitory site of Kir6.2. Numerous studies (58–63) have proposed such an interaction, but there is little direct evidence. Our model predicts that such an interaction is not necessary to explain the data.

How, then, might nucleotide occupancy at the NBSs be relayed to the pore? In the crystal structure of the ABC transporter McJD (64), the first cytosolic loop of TMD1 (and TMD2) contacts both NBDs, whereas the second interacts only with the NBD from the opposite monomer. It is possible the corresponding cytosolic loops (4,7) in SUR may form coupling helices that transmit conformational changes in the NBSs (produced by nucleotide binding/activation) to the TMDs. Both the cytoplasmic N-terminal (58,61,63) and C-terminal (60) regions of Kir6.2 have been proposed to interact with SUR. Likewise, many regions of SUR, including TMD0 of SUR1 (59), the loop connecting TMD0 to TMD1 of SUR1 (61), and the linker between TMD2 and NBD2 of SUR2A (62), have been suggested to modify gating of Kir6.2. To date, however, none of these interacting regions have been shown to directly transduce binding of Mg-nucleotides at SUR to gating changes at Kir6.2.

CONCLUSIONS

Although there has been much work on the structure and function of the NBSs of SUR and how they regulate K_{ATP} channel activity, our understanding is still far from complete. This is in part because there are few experiments in which nucleotide binding and ATPase activity have been measured in the intact K_{ATP} channel complex. The available results suggest that lack of Kir6.2, or the TMDs of SUR, influences nucleotide handling by the NBSs (compare (23) and (53)). Thus, it is hard to compare electrophysiological and biochemical data directly. A further problem is that SUR1 and SUR2 show differences in nucleotide handling—again, why they do so remains unclear. Although a high-resolution structure of the K_{ATP} channel, or even SUR, will undoubtedly provide many novel insights, it is unlikely to resolve the issue of precisely how MgATP binding/hydrolysis at NBS2 opens the channel. Functional data on nucleotide handling will still be required. We look forward to seeing how the results of such diverse approaches will combine to elucidate the working of the K_{ATP} channel, and hope that our (admittedly simple) model will provide testable predictions for experiments to come.

SUPPORTING MATERIAL

Two figures and one table are available at http://www.biophysj.org/biophysj/supplemental/S0006-3495(15)01100-5.

AUTHOR CONTRIBUTIONS

M.C.P. performed the modeling. N.V., F.M.A., and M.C.P. wrote the manuscript.

ACKNOWLEDGMENTS

We thank Peter Proks for a critical discussion and Chris Miller for helpful advice.

This work was supported by the European Research Council (grant 332620), The Wellcome Trust (grant 089795), and the Royal Society. F.M.A. holds a Royal Society/Wolfson merit award.

REFERENCES

1. Proks, P., and F. M. Ashcroft. 2009. Modeling K(ATP) channel gating and its regulation. *Prog. Biophys. Mol. Biol.* 99:7–19.
2. Gribble, F. M., S. J. Tucker, ..., F. M. Ashcroft. 1998. MgATP activates the beta cell KATP channel by interaction with its SUR1 subunit. *Proc. Natl. Acad. Sci. USA.* 95:7185–7190.
3. Nichols, C. G., S. L. Shyng, ..., J. Bryan. 1996. Adenosine diphosphate as an intracellular regulator of insulin secretion. *Science.* 272:1785–1787.
4. Shyng, S., T. Ferrigni, and C. G. Nichols. 1997. Regulation of KATP channel activity by diazoxide and MgADP. Distinct functions of the two nucleotide binding folds of the sulfonylurea receptor. *J. Gen. Physiol.* 110:643–654.
5. Tucker, S. J., F. M. Gribble, ..., F. M. Ashcroft. 1997. Truncation of Kir6.2 produces ATP-sensitive K+ channels in the absence of the sulphonylurea receptor. *Nature.* 387:179–183.
6. Inagaki, N., T. Gonoi, ..., J. Bryan. 1995. Reconstitution of IKATP: an inward rectifier subunit plus the sulfonylurea receptor. *Science.* 270:1166–1170.
7. Ashcroft, F. M. 2010. New uses for old drugs: neonatal diabetes and sulphonylureas. *Cell Metab.* 11:179–181.
8. Pearson, E. R., I. Flechtner, ..., A. T. Hattersley; Neonatal Diabetes International Collaborative Group. 2006. Switching from insulin to oral sulfonylureas in patients with diabetes due to Kir6.2 mutations. *N. Engl. J. Med.* 355:467–477.
9. Patel, D. J., H. J. Purcell, and K. M. Fox. 1999. Cardioprotection by opening of the K(ATP) channel in unstable angina. Is this a clinical manifestation of myocardial preconditioning? Results of a randomized

study with nicorandil. CESAR 2 investigation. Clinical European studies in angina and revascularization. *Eur. Heart J.* 20:51–57.

10. Aguilar-Bryan, L., C. G. Nichols, …, D. A. Nelson. 1995. Cloning of the beta cell high-affinity sulfonylurea receptor: a regulator of insulin secretion. *Science.* 268:423–426.
11. Davidson, A. L., E. Dassa, …, J. Chen. 2008. Structure, function, and evolution of bacterial ATP-binding cassette systems. *Microbiol. Mol. Biol. Rev.* 72:317–364.
12. Locher, K. P. 2009. Review. Structure and mechanism of ATP-binding cassette transporters. *Philos. Trans. R. Soc. Lond. B Biol. Sci.* 364:239–245.
13. Seyffer, F., and R. Tampé. 2015. ABC transporters in adaptive immunity. *Biochim. Biophys. Acta.* 1850:449–460.
14. ter Beek, J., A. Guskov, and D. J. Slotboom. 2014. Structural diversity of ABC transporters. *J. Gen. Physiol.* 143:419–435.
15. Jones, P. M., and A. M. George. 2012. Role of the D-loops in allosteric control of ATP hydrolysis in an ABC transporter. *J. Phys. Chem. A.* 116:3004–3013.
16. Urbatsch, I. L., K. Gimi, …, A. E. Senior. 2000. Investigation of the role of glutamine-471 and glutamine-1114 in the two catalytic sites of P-glycoprotein. *Biochemistry.* 39:11921–11927.
17. Matsuo, M., N. Kioka, …, K. Ueda. 1999. ATP binding properties of the nucleotide-binding folds of SUR1. *J. Biol. Chem.* 274:37479–37482.
18. Hohl, M., C. Briand, …, M. A. Seeger. 2012. Crystal structure of a heterodimeric ABC transporter in its inward-facing conformation. *Nat. Struct. Mol. Biol.* 19:395–402.
19. Hohl, M., L. M. Hürlimann, …, M. A. Seeger. 2014. Structural basis for allosteric cross-talk between the asymmetric nucleotide binding sites of a heterodimeric ABC exporter. *Proc. Natl. Acad. Sci. USA.* 111:11025–11030.
20. Li, J., K. F. Jaimes, and S. G. Aller. 2014. Refined structures of mouse P-glycoprotein. *Protein Sci.* 23:34–46.
21. Ward, A., C. L. Reyes, …, G. Chang. 2007. Flexibility in the ABC transporter MsbA: alternating access with a twist. *Proc. Natl. Acad. Sci. USA.* 104:19005–19010.
22. Masia, R., and C. G. Nichols. 2008. Functional clustering of mutations in the dimer interface of the nucleotide binding folds of the sulfonylurea receptor. *J. Biol. Chem.* 283:30322–30329.
23. Mikhailov, M. V., J. D. Campbell, …, F. M. Ashcroft. 2005. 3-D structural and functional characterization of the purified KATP channel complex Kir6.2-SUR1. *EMBO J.* 24:4166–4175.
24. Matsuo, M., K. Tanabe, …, K. Ueda. 2000. Different binding properties and affinities for ATP and ADP among sulfonylurea receptor subtypes, SUR1, SUR2A, and SUR2B. *J. Biol. Chem.* 275:28757–28763.
25. Bienengraeber, M., A. E. Alekseev, …, A. Terzic. 2000. ATPase activity of the sulfonylurea receptor: a catalytic function for the KATP channel complex. *FASEB J.* 14:1943–1952.
26. Matsuo, M., Y. Kimura, and K. Ueda. 2005. KATP channel interaction with adenine nucleotides. *J. Mol. Cell. Cardiol.* 38:907–916.
27. Horrigan, F. T., and R. W. Aldrich. 2002. Coupling between voltage sensor activation, Ca2+ binding and channel opening in large conductance (BK) potassium channels. *J. Gen. Physiol.* 120:267–305.
28. Craig, T. J., F. M. Ashcroft, and P. Proks. 2008. How ATP inhibits the open K(ATP) channel. *J. Gen. Physiol.* 132:131–144.
29. Drain, P., X. Geng, and L. Li. 2004. Concerted gating mechanism underlying KATP channel inhibition by ATP. *Biophys. J.* 86:2101–2112.
30. Antcliff, J. F., S. Haider, …, F. M. Ashcroft. 2005. Functional analysis of a structural model of the ATP-binding site of the KATP channel Kir6.2 subunit. *EMBO J.* 24:229–239.
31. Markworth, E., C. Schwanstecher, and M. Schwanstecher. 2000. ATP4-mediates closure of pancreatic beta-cell ATP-sensitive potassium channels by interaction with 1 of 4 identical sites. *Diabetes.* 49:1413–1418.
32. Enkvetchakul, D., and C. G. Nichols. 2003. Gating mechanism of KATP channels: function fits form. *J. Gen. Physiol.* 122:471–480.
33. Proks, P., H. de Wet, and F. M. Ashcroft. 2010. Activation of the K(ATP) channel by Mg-nucleotide interaction with SUR1. *J. Gen. Physiol.* 136:389–405.
34. Gribble, F. M., S. J. Tucker, and F. M. Ashcroft. 1997. The essential role of the Walker A motifs of SUR1 in K-ATP channel activation by Mg-ADP and diazoxide. *EMBO J.* 16:1145–1152.
35. Drain, P., L. Li, and J. Wang. 1998. KATP channel inhibition by ATP requires distinct functional domains of the cytoplasmic C terminus of the pore-forming subunit. *Proc. Natl. Acad. Sci. USA.* 95:13953–13958.
36. Masia, R., J. C. Koster, …, F. Barbetti. 2007. An ATP-binding mutation (G334D) in KCNJ11 is associated with a sulfonylurea-insensitive form of developmental delay, epilepsy, and neonatal diabetes. *Diabetes.* 56:328–336.
37. Dupuis, J. P., J. Revilloud, …, M. Vivaudou. 2008. Three C-terminal residues from the sulphonylurea receptor contribute to the functional coupling between the K(ATP) channel subunits SUR2A and Kir6.2. *J. Physiol.* 586:3075–3085.
38. Hopkins, W. F., S. Fatherazi, …, D. L. Cook. 1992. Two sites for adenine-nucleotide regulation of ATP-sensitive potassium channels in mouse pancreatic beta-cells and HIT cells. *J. Membr. Biol.* 129:287–295.
39. Proks, P., H. de Wet, and F. M. Ashcroft. 2014. Sulfonylureas suppress the stimulatory action of Mg-nucleotides on Kir6.2/SUR1 but not Kir6.2/SUR2A KATP channels: a mechanistic study. *J. Gen. Physiol.* 144:469–486.
40. Babenko, A. P. 2008. A novel ABCC8 (SUR1)-dependent mechanism of metabolism-excitation uncoupling. *J. Biol. Chem.* 283:8778–8782.
41. Hosy, E., and M. Vivaudou. 2014. The unusual stoichiometry of ADP activation of the KATP channel. *Front. Physiol.* 5:11.
42. Tammaro, P., C. Girard, …, F. M. Ashcroft. 2005. Kir6.2 mutations causing neonatal diabetes provide new insights into Kir6.2-SUR1 interactions. *EMBO J.* 24:2318–2330.
43. Zingman, L. V., A. E. Alekseev, …, A. Terzic. 2001. Signaling in channel/enzyme multimers: ATPase transitions in SUR module gate ATP-sensitive K+ conductance. *Neuron.* 31:233–245.
44. Zingman, L. V., D. M. Hodgson, …, A. Terzic. 2002. Tandem function of nucleotide binding domains confers competence to sulfonylurea receptor in gating ATP-sensitive K+ channels. *J. Biol. Chem.* 277:14206–14210.
45. Csanády, L., P. Vergani, and D. C. Gadsby. 2010. Strict coupling between CFTR's catalytic cycle and gating of its Cl- ion pore revealed by distributions of open channel burst durations. *Proc. Natl. Acad. Sci. USA.* 107:1241–1246.
46. Basso, C., P. Vergani, …, D. C. Gadsby. 2003. Prolonged nonhydrolytic interaction of nucleotide with CFTR's NH2-terminal nucleotide binding domain and its role in channel gating. *J. Gen. Physiol.* 122:333–348.
47. Tsai, M. F., M. Li, and T. C. Hwang. 2010. Stable ATP binding mediated by a partial NBD dimer of the CFTR chloride channel. *J. Gen. Physiol.* 135:399–414.
48. Vergani, P., S. W. Lockless, …, D. C. Gadsby. 2005. CFTR channel opening by ATP-driven tight dimerization of its nucleotide-binding domains. *Nature.* 433:876–880.
49. Ueda, K., N. Inagaki, and S. Seino. 1997. MgADP antagonism to Mg2+-independent ATP binding of the sulfonylurea receptor SUR1. *J. Biol. Chem.* 272:22983–22986.
50. Masia, R., D. Enkvetchakul, and C. G. Nichols. 2005. Differential nucleotide regulation of KATP channels by SUR1 and SUR2A. *J. Mol. Cell. Cardiol.* 39:491–501.
51. Matsuoka, T., K. Matsushita, …, Y. Kurachi. 2000. C-terminal tails of sulfonylurea receptors control ADP-induced activation and diazoxide modulation of ATP-sensitive K(+) channels. *Circ. Res.* 87:873–880.

52. Reimann, F., F. M. Gribble, and F. M. Ashcroft. 2000. Differential response of K(ATP) channels containing SUR2A or SUR2B subunits to nucleotides and pinacidil. *Mol. Pharmacol.* 58:1318–1325.

53. de Wet, H., M. V. Mikhailov, …, F. M. Ashcroft. 2007. Studies of the ATPase activity of the ABC protein SUR1. *FEBS J.* 274:3532–3544.

54. Masia, R., G. Caputa, and C. G. Nichols. 2007. Regulation of KATP channel expression and activity by the SUR1 nucleotide binding fold 1. *Channels (Austin).* 1:315–323.

55. de Wet, H., C. Fotinou, …, F. M. Ashcroft. 2010. The ATPase activities of sulfonylurea receptor 2A and sulfonylurea receptor 2B are influenced by the C-terminal 42 amino acids. *FEBS J.* 277:2654–2662.

56. Ortiz, D., L. Gossack, …, J. Bryan. 2013. Reinterpreting the action of ATP analogs on K(ATP) channels. *J. Biol. Chem.* 288:18894–18902.

57. Ortiz, D., P. Voyvodic, …, J. Bryan. 2012. Two neonatal diabetes mutations on transmembrane helix 15 of SUR1 increase affinity for ATP and ADP at nucleotide binding domain 2. *J. Biol. Chem.* 287:17985–17995.

58. Babenko, A. P., and J. Bryan. 2002. SUR-dependent modulation of KATP channels by an N-terminal KIR6.2 peptide. Defining intersubunit gating interactions. *J. Biol. Chem.* 277:43997–44004.

59. Chan, K. W., H. Zhang, and D. E. Logothetis. 2003. N-terminal transmembrane domain of the SUR controls trafficking and gating of Kir6 channel subunits. *EMBO J.* 22:3833–3843.

60. Lodwick, D., R. D. Rainbow, …, R. I. Norman. 2014. Sulfonylurea receptors regulate the channel pore in ATP-sensitive potassium channels via an intersubunit salt bridge. *Biochem. J.* 464:343–354.

61. Pratt, E. B., Q. Zhou, …, S. L. Shyng. 2012. Engineered interaction between SUR1 and Kir6.2 that enhances ATP sensitivity in KATP channels. *J. Gen. Physiol.* 140:175–187.

62. Rainbow, R. D., M. James, …, R. I. Norman. 2004. Proximal C-terminal domain of sulphonylurea receptor 2A interacts with pore-forming Kir6 subunits in KATP channels. *Biochem. J.* 379:173–181.

63. Reimann, F., S. J. Tucker, …, F. M. Ashcroft. 1999. Involvement of the n-terminus of Kir6.2 in coupling to the sulphonylurea receptor. *J. Physiol.* 518:325–336.

64. Choudhury, H. G., Z. Tong, …, K. Beis. 2014. Structure of an antibacterial peptide ATP-binding cassette transporter in a novel outward occluded state. *Proc. Natl. Acad. Sci. USA.* 111:9145–9150.

The nucleotide-binding sites of SUR1: a mechanistic model (Supporting Material)

Natascia Vedovato, Frances M Ashcroft and Michael C Puljung

Department of Physiology, Anatomy and Genetics
University of Oxford
Parks Road
Oxford OX1 3PT

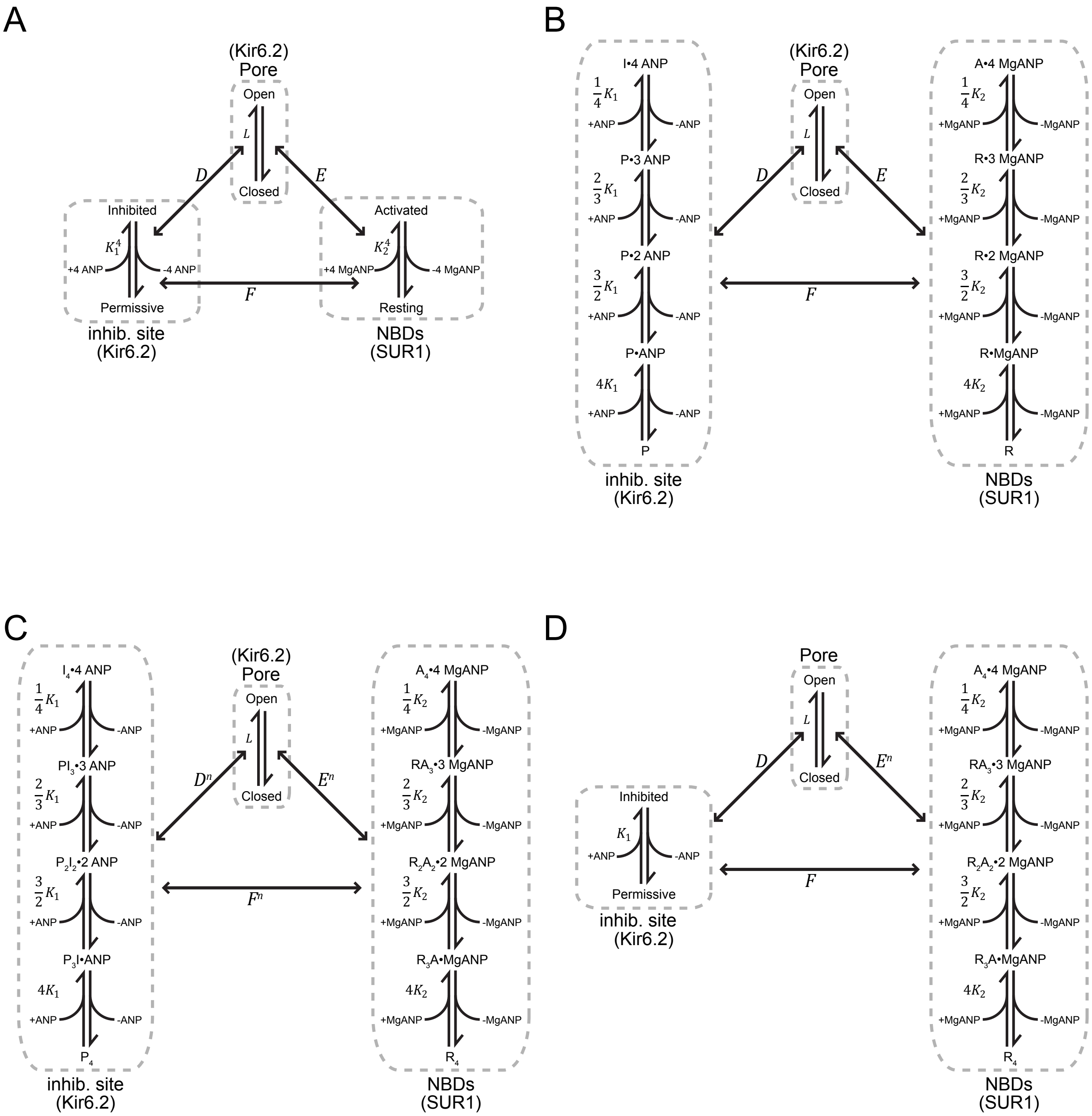
A
(Kir6.2)
Pore
Open
Closed
L
D
E
Inhibited
Permissive
+4 ANP
-4 ANP
inhib. site
(Kir6.2)
Activated
Resting
+4 MgANP
-4 MgANP
NBDs
(SUR1)
F
B
I•4 ANP
P•3 ANP
P•2 ANP
P•ANP
P
+ANP
-ANP
A•4 MgANP
R•3 MgANP
R•2 MgANP
R•MgANP
R
+MgANP
-MgANP
C
D^n
E^n
F^n
I4•4 ANP
PI3•3 ANP
P2I2•2 ANP
P3I•ANP
P4
A4•4 MgANP
RA3•3 MgANP
R2A2•2 MgANP
R3A•MgANP
R4
D
Pore

Figure S1 Alternative gating models. (A) Cooperative model. Schematic representing the gating of K_{ATP} channels as three interacting domains, the pore, the inhibitory nucleotide-binding sites on four Kir6.2 subunits, and the NBSs of four SUR1 subunits. Nucleotide binding to SUR or Kir6.2 is cooperative and results in a concerted conformational change that affects channel gating.

$$P_o = \frac{L(K_1^4[ANP]^4D+1)(K_2^4[ANP]^4E+1)}{(K_1^4[ANP]^4+1)(K_2^4[ANP]^4+1)+L(K_1^4[ANP]^4D+1)(K_2^4[ANP]^4E+1)}$$

(B) Sequential model. Gating schematic in which nucleotides bind independently to each SUR1 or each Kir6.2 subunit. Upon binding of the fourth nucleotide to either domain, there is a concerted conformational change in all four subunits that affects channel gating. The states of the Kir6.2 binding sites are designated "P" for "permissive" and "I" for "Inhibited." The states of the NBDs are labeled "R" for "resting" and "I" for "activated."

$$P_o = \frac{L(K_1^4[A]^4D+4K_1^3[A]^3+6K_1^2[A]^2+4K_1[A]+1)(K_2^4[A]^4E+4K_2^3[A]^3+6K_2^2[A]^2+4K_2[A]+1)}{(K_1[A]+1)^4(K_2[A]+1)^4+L(K_1^4[A]^4D+4K_1^3[A]^3+6K_1^2[A]^2+4K_1[A]+1)(K_2^4[A]^4E+4K_2^3[A]^3+6K_2^2[A]^2+4K_2[A]+1)}$$

(C) MWC-type model. Model in which nucleotides bind to each of the four subunits of SUR1 and each of the four subunits of Kir6.2 independently and each binding event contributes energetically equally to the opening of the pore (by a factor of E for each bound SUR subunit and a factor of D for each ANP-bound Kir6.2). Subscripts indicate the number of subunits in either state.

$$P_o = \frac{L(K_1[ANP]D+1)^4(K_2[ANP]E+1)^4}{(K_1[ANP]+1)^4(K_2[ANP]+1)^4+L(K_1[ANP]D+1)^4(K_2[ANP]E+1)^4}$$

(D) Mixed model. Mixed gating model in which the NBDs each bind nucleotide independently and make equal contributions to the pore and the inhibitory Kir6.2 binding site behaves as a single site.

$$P_o = \frac{L(K_1[ANP]D+1)(K_2[ANP]E+1)^4}{(K_1[ANP]+1)(K_2[ANP]+1)^4+L(K_1[ANP]D+1)(K_2[ANP]E+1)^4}$$

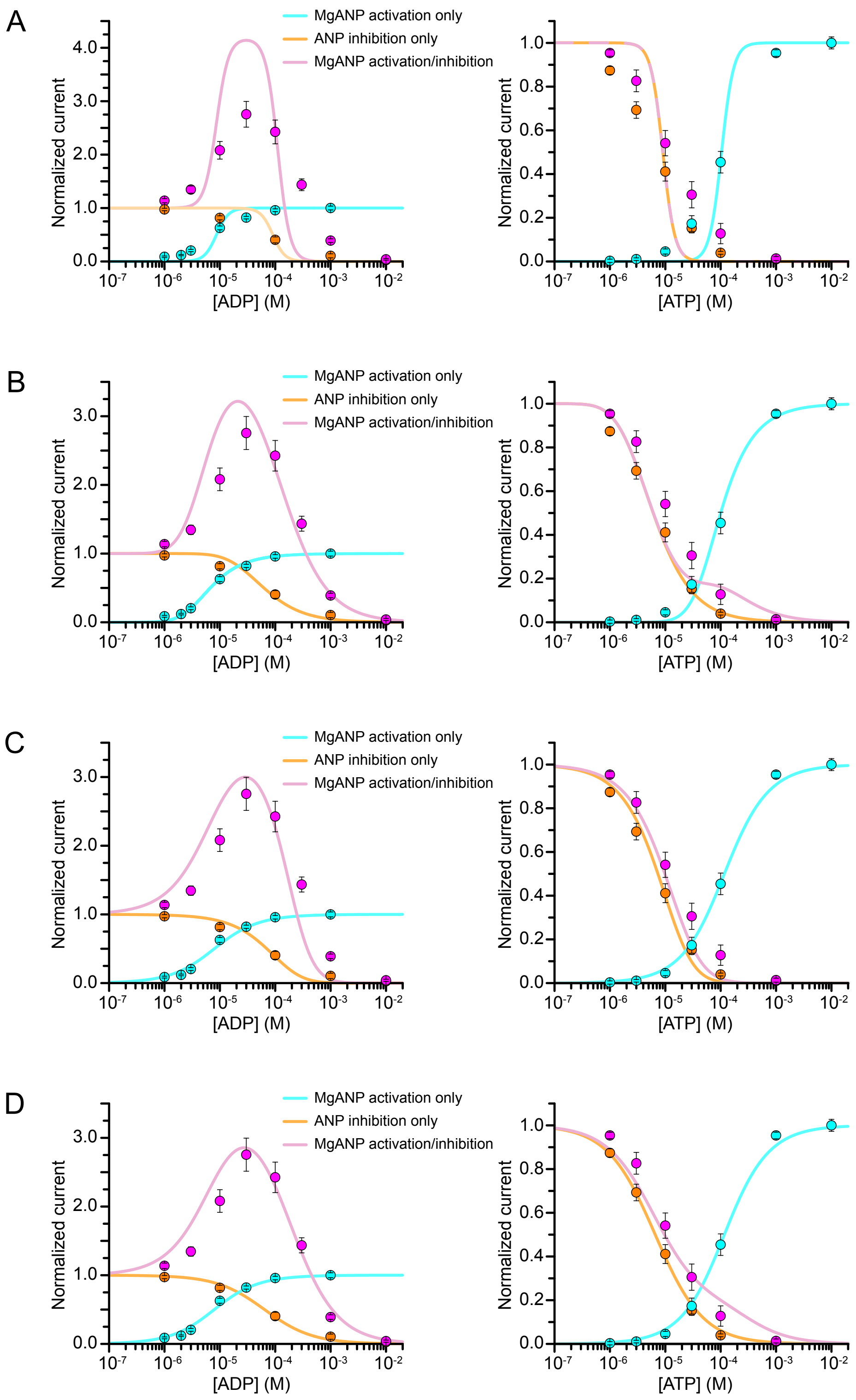
A
B
C
D
MgANP activation only
ANP inhibition only
MgANP activation/inhibition
Normalized current
[ADP] (M)
[ATP] (M)
10-7
10-6
10-5
10-4
10-3
10-2
0.0
1.0
2.0
3.0
4.0
0.2
0.4
0.6
0.8

Figure S2 Fits to alternative gating models (A) Fits of the cooperative scheme from Fig. S1A to data from Proks *et al.* (33). (B) Fits of the sequential scheme from Fig. S1B to data from Proks *et al.* (33). (C) Fits of the MWC-type scheme from Fig. S1C to data from Proks *et al.* (33). (D) Fits of the mixed gating scheme from Fig. S1D to data from Proks *et al.* (33). All values and RMSDs are reported in Table S1.

Table S1 Values and RMSD for K_{ATP} gating models

	**Single site**		**Cooperative**		**Sequential**		**MWC**		**Mixed**	
	**ADP**	**ATP**	**ADP**	**ATP**	**ADP**	**ATP**	**ADP**	**ATP**	**ADP**	**ATP**
K_1 (M^{-1})	1.8×10^{4}	1.8×10^{5}	1.1×10^{4}	1.2×10^{5}	9.6×10^{4}	9.9×10^{5}	3.2×10^{3}	3.0×10^{4}	1.8×10^{4}	1.8×10^{5}
K_2 (M^{-1})	5.4×10^{4}	3.6×10^{3}	9.4×10^{4}	7.8×10^{3}	4.3×10^{5}	2.9×10^{4}	1.2×10^{5}	8.5×10^{3}	1.2×10^{5}	8.5×10^{3}
L	0.18	0.18	0.18	0.18	0.18	0.18	0.18	0.18	0.18	0.18
D	1×10^{-6}	1×10^{-6}	1×10^{-6}	1×10^{-6}	1×10^{-6}	1×10^{-6}	0.032*	0.032*	1×10^{-6}	1×10^{-6}
E	9.5	9.5	9.5	9.5	9.5	9.5	1.76†	1.76†	1.76†	1.76†
**RMSD**	0.15	0.04	0.49	0.13	0.23	0.06	0.24	0.05	0.15	0.04

*$D^4 = 1 \times 10^{-6}$
†$E^4 = 9.5$
